# Association Between Dietary Intakes and Plaque Vulnerability Measured by Optical Coherence Tomography in Patients With Coronary Heart Disease: A Mediation Analysis of Inflammatory Factors

**DOI:** 10.3389/fnut.2022.920892

**Published:** 2022-06-14

**Authors:** Ling Li, Zhenjuan Zhao, Yini Wang, Xueqin Gao, Guojie Liu, Bo Yu, Ping Lin

**Affiliations:** Department of Cardiology, The Second Affiliated Hospital of Harbin Medical University, Harbin, China

**Keywords:** coronary heart disease, plaque vulnerability, coronary atherosclerosis, optical coherence tomography, diet, nutrient, inflammation, inflammatory cytokine

## Abstract

Although studies have proven that diet has a critical role in preventing or delaying atherosclerosis and is far simpler to adjust and adhere to than other risk factors, the underlying mechanisms behind this effect remain not well comprehended. The purpose of this investigation was to determine the impact of inflammatory factors on the connection between dietary ingestion and coronary plaque fragility as measured via optical coherence tomography (OCT) in patients with coronary heart disease (CHD). This research eventually comprised 194 participants with CHD who met the inclusion and exclusion criteria. Semi-quantitative food frequency questionnaire (SQFFQ) was utilized to investigate dietary consumption status, serum levels of inflammatory biomarkers were analyzed using enzyme-linked immunosorbent assay, and OCT was employed to identify the plaque susceptibility of causative lesions in the body. Following correction for statistically meaningful possible confounders in univariate analysis, quartiles of soy and nuts, fruits and vitamin C were negatively associated with coronary plaque vulnerability. Conversely, the upper quartile group of sodium intake had 2.98 times the risk of developing vulnerable plaques compared with the most minimal quartile group. Meanwhile, we observed an inverse dose–response connection between vitamin C consumption and inflammatory biomarkers as well as plaque vulnerability. More importantly, tumor necrosis factor- α (TNF-α) and interleukin-6 (IL-6) were significant mediators of the connection between vitamin C and plaque vulnerability, suggesting that vitamin C may inhibit the atherosclerotic inflammatory process by decreasing the expression of IL-6 and TNF-α, thereby reducing the risk of vulnerable plaques. These new findings provide crucial clues to identify anti-inflammatory dietary components as effective therapeutic approaches in the management of CHD, while also providing some insights into their mechanisms of action.

## Introduction

For despite improvements in interventional and pharmacological therapy for CHD, it is still the primary reason of mortality and disability-adjusted life years (DALYs) worldwide (1). Obstructive atherosclerotic plaque produces a restriction in blood supply to the myocardium, causing CHD (2). It is indicated by robust evidence that CHD evolvement has resulted by the pivotal mechanism of coronary plaque vulnerability, and vulnerable plaque which is distinguished by a large lipid necrotic core and thin fibrous cap is prone to rupture and lead to coronary thrombosis. OCT is a catheter-based imaging technique that uses near-infrared light to obtain cross-sectional images of the coronary arteries. OCT is enabled to evaluate many morphological characteristics of coronary atherosclerosis *in vivo* due to its unusually high resolution (10–20 m) (3). Clinical findings revealed that OCT-detected vulnerable plaque was inextricably linked to a higher chance of developing major adverse cardiac events (MACE) (4–6). Therefore, effective measures to control and reduce plaque vulnerability are essential to reduce the poor prognosis of CHD.

Among various identified risk factors for CHD, food and eating habits plays a critical influence and are preventable factors (7). Many research have increased our knowledge about the association between nutritional intake and heart health during the last few decades. Increased consumption of vegetables and fruits, nuts, antioxidant vitamins, folate, and dietary fiber may be preventive factors for the incidence and development of CHD, according to prospective studies and meta-analyses (8–13). On the other side, increased consumption of cholesterol, eggs, red meat, processed meat and sugar-sweetened drinks may raise the chance and development of CHD (14, 15). A good diet has also been linked to a decreased incidence of MACE in big populations (16). In patients with CHD treated with percutaneous coronary intervention (PCI), poor consumption of fruits (OR = 0.30, 95% CI = 0.12–0.68) and vegetables (OR = 0.48, 95% CI = 0.27–0.91) are both independent risk factors for in-stent restenosis (ISR) (17). Further research using OCT to evaluate plaque vulnerability, we have found that salt and sodium are dietary components that contribute to plaque vulnerability, while vegetables, fruits, dietary fiber, folate and vitamin C have been revealed to be dietary preventive factors (18). However, the mechanism between dietary intake and plaque vulnerability is unclear.

In fact, atherosclerosis is an inflammatory process, with inflammatory pathways present throughout the entire process, from the onset and progression of atherosclerosis to plaque rupture, erosion, and thrombus formation (19). Inflammatory variables such as high-sensitivity C-reactive protein (hs-CRP), TNF-α, and IL-6 are closely connected to plaque lipid core enlargement and fibrous cap breakdown, suggesting that inflammation and immune cell activation play a key role in defining plaque vulnerability (20–22). At the same time, earlier study has established a clear correlation between inflammation and the overall diet and specific dietary components (23, 24). Fruits and vegetables have been demonstrated to lower inflammation, whereas red meat intake has been shown to promote it (25, 26). Consuming more healthful and anti-inflammatory food ingredients like fruits and green leafy vegetables while reducing consumption of pro-inflammatory foods like processed meat and sugar-sweetened beverages, according to a recent meta-analysis, may be crucial in lowering the risk of CHD and associated mortality (27). Following the Mediterranean diet (MeDiet) decreases plasma levels of inflammatory cytokines linked with various phases of atheroma plaque formation in old age patients with a high propensity to CHD, according to a five-year multicenter randomized controlled experiment in Spain (28). Miller et al. reported that increasing dietary fiber consumption resulted in considerably reduced plasma indicators of inflammatory state (29). A considerably larger study from the United Kingdom discovered a substantial inverse linear relationship between fiber consumption and hs-CRP (30). Despite this, few studies have been done to see if inflammatory variables play a role in the relationship between dietary intake and coronary plaque vulnerability.

Consequently, our objectives were to: (i) investigate the link between dietary intakes and plaque vulnerability; and (ii) identify inflammatory mediators and their impact on the relationship between food nutrients and coronary plaque vulnerability.

## Materials and Methods

### Study Participants

**FIGURE 1:**
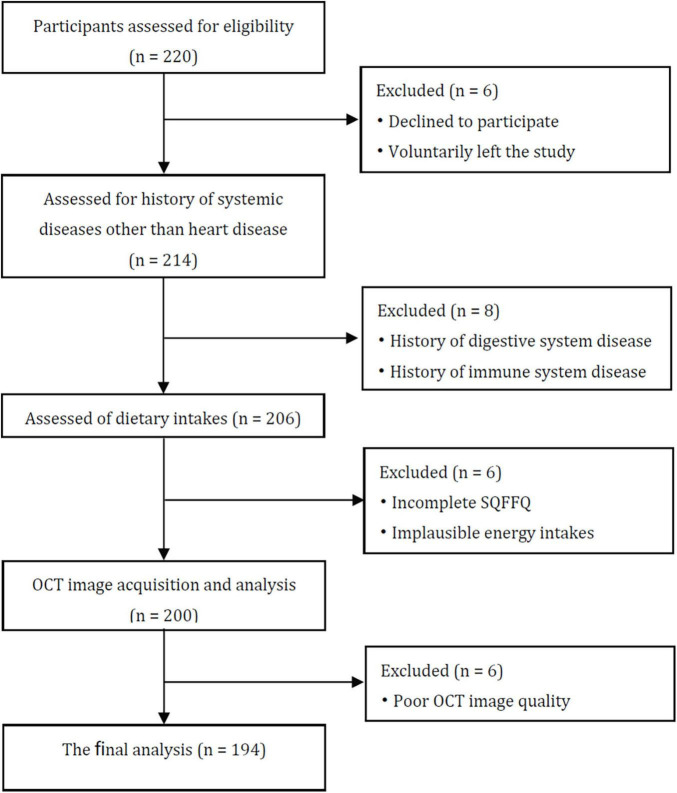
Consort flow diagram. SQFFQ, Semi-quantitative food frequency questionnaire; OCT, Optical coherence tomography.

This study was a cross-sectional design, and used non-probabilistic sampling. From March 2020 to September 2021, 220 CHD patients were recruited to undergo OCT tomography at Harbin Medical University’s Second Hospital Affiliated. The participants were between the ages of 20 and 75. The research excluded patients with current infection, obvious cognitive deficits, mental comorbidity, long-term anti-inflammatory medication usage, left ventricular ejection fraction (LVEF) < 40% and individuals with allergy, rheumatoid disease, or malignancy. In total, 26 participants were excluded based on the exclusion criteria. Thus, 194 individuals with CHD were eventually enrolled throughout this research (Figure 1).

### Study Procedures

After informed consent was obtained, these participants were invited to fill questionnaires prior to conducting OCT evaluations. Highly qualified investigators offered in-depth explanations of the surveys to the subjects, and provided necessary help for participants to complete the questionnaire smoothly. During the OCT examination, blood samples were taken. Meantime, sociodemographic and medical information was extracted from their clinical documents and, if required, verified by subjects. Prior to actually participating in the research, every individuals provided written informed permission for participation. The Harbin Medical University (China) Human Ethics Review Committee approved the study (protocol code ky2020-058, approval date March 20, 2020), which was carried out in accordance with the Helsinki Declaration.

### Assessment of Demographic and Clinical Data

Among the information gathered were: gender, age, body mass index (BMI), diagnosis of acute myocardial infarction, familial CHD, hypertension (systolic pressure ≥ 140 mmHg or diastolic pressure ≥ 90 mmHg on two separate occasions, or whether the patient was using anti-hypertensive medications) (31), hyperlipidemia (blood total cholesterol level ≥ 5.7 mmol/L and/or triglycerides concentration ≥ 1.7 mmol/L) (32), diabetes mellitus (DM; fasting plasma glucose level ≥ 7.0 mmol/L, a random venous plasma glucose level ≥ 11.1 mmol/L, 2-h plasma glucose concentration ≥ 11.1 mmol/L following an oral glucose tolerance test, or any combination of these) (33), smoking status (1 cigarette each day or more than 100 cigarettes consumed in an entire life) (34) and drinking condition (a daily alcohol intake of ≥ 24 g) (35).

Fasting plasma glucose (FPG), total cholesterol (TC), triglyceride (TG), low-density lipoprotein cholesterol (LDL-C), and high-density lipoprotein cholesterol (HDL-C) were among the clinical lab indicators examined and collected. The location of the assay was the Department of Clinical Laboratory, Harbin Medical University’s Second Hospital Affiliated.

### Assessment of Dietary Intakes

Prior conducting this inquiry, every personnel participating inside this investigation received training in the relevant research procedures. The SQFFQ, which was created and verified for the Chinese, was used to assess food consumption and consumption frequency of the study samples. The SQFFQ included 3 parts: food checklist, frequency of food consumption, and weight for every item consumed. Grain and potatoes, legumes and nuts, vegetables, fruits, domestic animals and poultry, milk, eggs, fish and shrimp, oils and salt were the ten categories in this survey. Subjects recorded their average intake frequency per food over the course of a year on an eight-point scale ranging from “almost never” to “3 times one day or more” (visualized utilizing prevalent container and weight units, for instance, bowls, cups, and spoons). Their mean quotidian consumption for every foodstuff was obtained via computing weighted frequency everyday and consumption amount per unit, then normalized to gram/day. The Chinese version of the SQFFQ has been validated and can be used to classify study subjects according to the food or nutrient intake during one year (36). The Food Nutrition Calculator V2.7.3 from the China CDC Nutrition and Food Safety Institute was used to calculate daily dietary nutrient consumption.

### Markers of Inflammation Cytokines Measurement

At the time of OCT checking, fresh venous circulating blood sample (5 mL) was taken from each patient in order to test the serum levels of inflammatory cytokines. Afterward, a 15-min centrifugation at 3,000 rpm and 4°C was performed on these test samples, aliquots were then kept in reserve at –80°C until further analysis was necessary. TNF-α, IL-6, and hs-CRP concentrations were measured using an enzyme-linked immunosorbent assay, with analytic sensitivities of 4.69, 4.69, and 8.00 pg/ml, respectively. Blind method analysis of a single lot was performed. The aforementioned three inflammatory biomarkers had less than 10% inter- and intra-assay coefficients of variation.

### Optical Coherence Tomography Image Acquisition and Analysis

**FIGURE 2:**
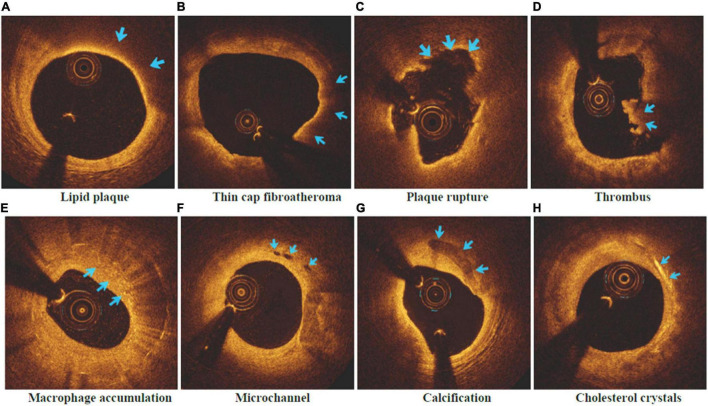
Vulnerable plaque and related characteristics in OCT images. **(A)** Lipid plaque; **(B)** Thin cap fibroatheroma; **(C)** Plaque rupture; **(D)** Thrombus; **(E)** Macrophage accumulation; **(F)** Microchannel; **(G)** Calcification; **(H)** Cholesterol crystals.

The intracoronary frequency- or time-domain OCT system (C7XR system, Saint Jude Medical, Westford, MA, United States) was used in this study to do OCT imaging (37). Offline software was utilized to capture and evaluate pictures of coronary arteries bearing culprit plaques. Two seasoned professionals completed the review of all OCT photographs respectively following the standards of OCT Clinical Expert Consensus Statement, and they were not privy to the participant’s electronic healthcare data. Once the conclusions of the two evaluators were inconsistent, the arbitration and compromise was conducted by one additional researcher. The following OCT imaging markers (37) were assessed: Lipid plaques (low signal region with diffuse boundary) and fibrous plaques (homogeneous high-backscattering region) were separated. Lipid arcs of lipid-rich plaques (maximum lipid arc > 2 quadrants) were recorded at 1-mm intervals throughout the lesion, with an average value of the thinnest region tested three times utilized as the fibrous cap thickness parameter. As shown in Figure 2, a lipid plaque with a lipid arc of ≥ 90° and a fibrous cap thickness of < 65 μm was designated as thin cap fibroatheroma (TCFA) in this study as the specific indication utilized to detect plaque vulnerability (38). Fibrous cap discontinuities and plaque cavities were signs of plaque rupture (39). The thrombus presented with an irregular lump extending into an arterial lumen of at least 250 μm in diameter; Macrophage accumulation was characterized by a rise in signal strength inside of plaque, together with the presence of heterogeneous backward shadows; Microchannel showed as speckled black rounded areas with diameter between 50 and 300 μm inside the plaque, and appeared in more than three consecutive frames; Calcification was described as an area with low backscattering signal and a sharp border. Cholesterol crystals were defined as linear, highly backscattering structures within the plaque (40).

### Statistical Analysis

IBM SPSS Statistics was used to conduct statistical analysis (V.26.0, IBM Corp, Armonk, NY, United States). A statistically significant two-sided *p* value of 0.05 was used. Before analysis, variables that were not normally distributed were natural-logarithmically converted to achieve near normality, as established by the Kolmogorov-Smirnov test. When applicable, continuous data is provided as the mean (standard deviation), or median (25th, 75th) and evaluated using Student’s *t*-test or non-parametric test (Mann–Whitney *U* test). Categorical variables are reported as a percentage, and chi-square tests were used to compare groups. To correspond to a normal distribution, data for inflammatory biomarkers were standardized using log transformations.

The correlations between food and nutrient intakes and all inflammatory biomarkers and atherosclerotic plaque features factors were investigated using the Spearman correlation analysis. The connection of food and nutrient intakes with atherosclerotic plaque features measured by OCT was investigated using logistic regression analysis. The dependent variable was susceptible plaque characteristics, the independent variables were quartiles of food and nutrient intakes that gave a *p* value < 0.1 in univariate analysis, and any confounding variables that were statistically significant in univariate analysis were adjusted for. For food and nutrient intake categories, multivariable logistic regression models were used to estimate the odds ratio (OR) and 95% confidence interval (CI) of plaque vulnerability, with the lowest intake grouping as the reference. The quartiles median value was used as a quasi-continuous variable in the model to calculate *p* values for trends.

**FIGURE 3:**
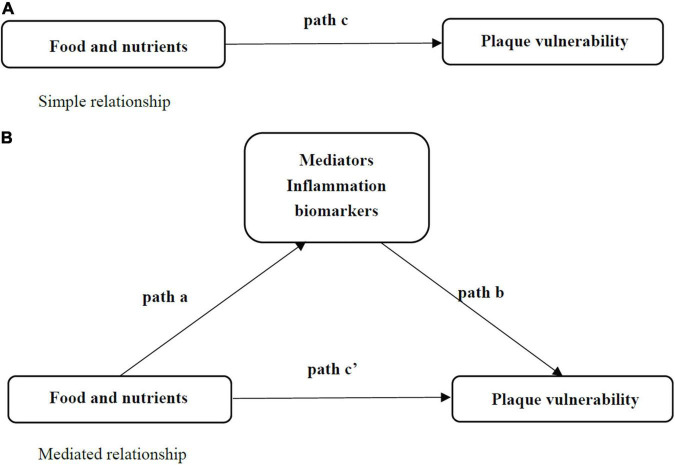
Simple relationship and Mediated relationship. Non-Mediated pathways **(A)** and Mediated pathways **(B)** between food and nutrients and plaque vulnerability. Path *c*’ represents the direct effect of food and nutrients on plaque vulnerability with the mediator included in the model. The indirect effect is the product of path *a* and path *b* (path a*b). Each mediator was considered in a separate statistical model.

This study evaluated the mediating effects of inflammatory biomarkers on the link between food and nutrient intakes and plaque vulnerability using Hayes’ (41) PROCESS tool of model 4 (42). As shown in Figure 3, bootstrap mediation analyses were performed. In the absence of mediating variables, the bootstrap analyses made it easier to assess the direct association between food and nutrient intakes and plaque vulnerability (path *c*; simple relationship in Figure 3A). Following that, a link was found between food and nutrient intakes and each of the suggested mediators (path *a*), as well as each mediator and plaque vulnerability (path *b*). The effect of food and nutrient intakes on plaque vulnerability by TNF-α, IL-6 was defined as an indirect effect (path a*b) in the mediation analysis. After correcting for inflammatory indicators, a direct effect (path *c*’) was defined as the effect of food and nutrient intakes on plaque vulnerability (Mediated relationship in Figure 3B). The mediation analysis was carried out, using 5,000 bootstrap samples. The mediating impact was judged significant if the 95% confidence interval for an effect did not include a zero.

## Results

### Patient Characteristics and Laboratory Results

A total of 194 patients were enrolled in the project, with an average age of 56.08 ± 10.99 years. Patients were split into two groups based on whether or not TCFA occurred: the vulnerable plaque group (98, 50.5%) and the non-vulnerable plaque group (96, 49.5%). Table 1 shows that a higher prevalence of myocardial infarction was found (*p* = 0.015) in the vulnerable plaque group, and the TG, TC and LDL-C levels were significantly higher (*p* = 0.030, *p* < 0.001, and *p* < 0.001, respectively) than the non-vulnerable plaque group. The vulnerable plaque group had greater levels of tumor necrosis factor-α (TNF-α) and interleukin-6 (IL-6) in the laboratory than the non-vulnerable plaque group (both of *p* = 0.001).

**TABLE 1:** Characteristics of vulnerable plaque and non-vulnerable plaque individuals (*N* = 194).

Variables	Total sample (*n* = 194)	Vulnerable plaque (*n* = 98)	Non-vulnerable plaque (*n* = 96)	Test value	*p*-value
Age [years, M(SD)]	56.08 (10.99)	56.26 (11.45)	55.90 (10.55)	*t* = 0.227	0.821
BMI [kg/m^2^, M(SD)]	24.91 (3.89)	24.91 (3.77)	24.91 (4.02)	*t* = -0.001	0.999
Male (*n*,%)	114 (58.8%)	63 (64.3%)	51 (53.1%)	χ2 = 2.493	0.114
Smoking history (*n*,%)	115 (59.3%)	58 (59.2%)	57 (59.4%)	χ2 = 0.001	0.978
Drinking history (*n*,%)	88 (45.4%)	48 (49.0%)	40 (41.7%)	χ2 = 1.046	0.306
Hypertension (*n*,%)	79 (40.7%)	39 (39.8%)	40 (41.7%)	χ2 = 0.070	0.791
Diabetes mellitus (*n*,%)	30 (15.5%)	15 (15.3%)	15 (15.6%)	χ2 = 0.004	0.951
Hyperlipidemia (*n*,%)	29 (14.9%)	12 (12.2%)	17 (17.7%)	χ2 = 1.139	0.286
Family history of CHD (*n*,%)	50 (25.8%)	27 (27.6%)	23 (24.0%)	χ2 = 0.327	0.567
Myocardial infarction (*n*,%)	182 (93.8%)	96 (98.0%)	86 (89.6%)	χ2 = 5.863	0.015
*Laboratory findings*					
Blood glucose [mmol/L, M(IQR)]	6.64 (5.65, 8.06)	6.58 (5.69, 7.99)	6.78 (5.54, 8.16)	*z* = 0.104	0.918
Log TG [mmol/L, M(SD)]	0.14 (0.25)	0.17 (0.27)	0.10 (0.23)	*t* = −2.187	0.030
Log TC [mmol/L, M(SD)]	0.65 (0.12)	0.69 (0.13)	0.62 (0.09)	*t* = 4.841	< 0.001
Log HDL-C [mmol/L, M(SD)]	0.08 (0.12)	0.09 (0.12)	0.07 (0.12)	*t* = 0.909	0.364
Log LDL-C [mmol/L, M(SD)]	0.45 (0.14)	0.50 (0.12)	0.39 (0.13)	*t* = 6.157	< 0.001
*Inflammatory biomarkers*					
Log TNF-α [ng/L, M(SD)]	1.05 (0.15)	1.09 (0.16)	1.02 (0.13)	*t* = 3.495	0.001
Log IL-6 [pg/ml, M(SD)]	1.30 (0.27)	1.37 (0.28)	1.24 (0.26)	*t* = 3.509	0.001
Log hs-CRP [mg/L, M(SD)]	0.62 (0.42)	0.64 (0.41)	0.61 (0.44)	*t* = 0.582	0.561

*M(SD), mean (standard deviation); M(IQR), median (interquartile range); BMI, body mass index; CHD, coronary heart disease; TC, total cholesterol; TG, triglyceride; LDL-C, low density lipoprotein cholesterol; HDL-C, high-density lipoprotein cholesterol; TNF-α, tumor necrosis factor-α; IL-6, interleukin-6; hs-CRP, high sensitivity C-reactive protein; Log, logarithmical.*

### Optical Coherence Tomography Findings

The OCT findings of the two groups were shown in Table 2. When compared to the non-vulnerable plaque group, the vulnerable plaque group had a longer lipid core, thinner fibrous cap thickness, and bigger lipid arc (all *p* < 0.001). Meanwhile, patients with susceptible plaques had a higher rate of plaque rupture, macrophage infiltration, thrombus, and cholesterol crystal formation than those with non-vulnerable plaques (all *p* < 0.001). Furthermore, susceptible plaques exhibited a greater rate of calcification and microchannels (*p* = 0.006 and *p* = 0.022, respectively).

**TABLE 2:** OCT characteristics of vulnerable plaque and non-vulnerable plaque individuals (*N* = 194).

Characteristics	Total sample (*n* = 194)	Vulnerable plaque (*n* = 98)	Non-vulnerable plaque (*n* = 96)	Test value	*p*-value
*Quantitative variable*					
Plaque length [mm, M(IQR)]	9.50 (3.18, 15.58)	12.35 (7.88, 19.28)	5.45 (1.40, 10.80)	*z* = 5.938	< 0.001
Fibrous cap thickness [μm, M(IQR)]	56.67 (40.00, 70.00)	43.33 (33.33, 53.33)	70.00 (64.91, 93.33)	*z* = -10.497	< 0.001
Lipid arc [deg, M(SD)]	137.34 (83.73)	194.68 (55.38)	78.81 (65.25)	*t* = 13.346	< 0.001
*Qualitative variable*					
Rupture (*n*,%)	122 (62.9%)	89 (90.8%)	33 (34.4%)	χ2 = 66.191	< 0.001
Lipid plaque (*n*,%)	128 (66.0%)	93 (94.9%)	35 (36.5%)	χ2 = 73.784	< 0.001
Macrophage infiltration (*n*,%)	144 (74.2%)	84 (85.7%)	60 (62.5%)	χ2 = 13.661	< 0.001
Thrombus (*n*,%)	164 (84.5%)	94 (95.9%)	70 (72.9%)	χ2 = 19.627	< 0.001
Calcification (*n*,%)	106 (54.6%)	63 (64.3%)	43 (44.8%)	χ2 = 7.436	0.006
Microchannel (*n*,%)	101 (52.1%)	59 (60.2%)	42 (43.8%)	χ2 = 5.261	0.022
Cholesterol crystal (*n*,%)	127 (65.5%)	83 (84.7%)	44 (45.8%)	χ2 = 32.392	< 0.001

*M(IQR), median (interquartile range); M(SD), mean (standard deviation).*

### Multivariate Regression Analysis for the Correlations of Dietary Intakes With Plaque Vulnerability

The baseline nutritional intake of the study participants was summarized in Table 3. The study participants were divided into four groups based on their quartile levels of daily food and nutrient intake, which generated a *p* value < 0.1 in univariate analysis (Table 3). To find relationships with susceptible plaque, researchers used univariate and multivariable logistic regression models with the lowest intake group as a reference. A backward stepwise multivariable logistic regression model with admission criteria of *p* < 0.05 and removal criteria of *p* > 0.1 was used to include factors identified significant in univariate analysis and important variables for professional consideration. After adjusting for gender, age, body mass index, history of myocardial infarction, TC, TG and LDL-C, Table 4 revealed that rising quartiles of dietary consumption of soy and nuts, fruits, and vitamins C remained independently linked with lower risk of susceptible plaque (*p* for trend with adjustment: 0.028, 0.002, and 0.003, respectively).

**TABLE 3:** Dietary intake status of vulnerable plaque and non-vulnerable plaque patients (*N* = 194).

Variables	Total Sample (*n* = 194)	Vulnerable plaque (*n* = 98)	Non-vulnerable plaque (*n* = 96)	Test value	*p*-value
Cereal and potatoes [g/d, M(SD)]	504.79 (151.79)	519.04 (162.03)	490.23 (139.92)	*t* = 1.324	0.187
Soy and nuts [g/d, M(IQR)]	9.99 (3.33, 24.58)	6.67 (2.92, 21.19)	13.33 (3.33, 31.71)	*z* = −2.077	0.038
Vegetables [g/d, M(IQR)]	336.96 (256.94, 472.08)	312.54 (230.63, 425.06)	361.52 (273.87, 513.89)	*z* = -2.678	0.007
Fruits [g/d, M(IQR)]	161.32 (69.95, 283.54)	132.53 (55.06, 229.78)	216.50 (112.10, 330.04)	*z* = -3.777	<0.001
Livestock and poultry [g/d, M(IQR)]	63.34 (28.04, 120.01)	59.99 (27.68, 120.13)	67.26 (28.70, 119.47)	*z* = -0.645	0.519
Milk [g/d, M(IQR)]	0.00 (0.00, 64.28)	0.00 (0.00, 52.68)	0.00 (0.00, 89.29)	*z* = -0.630	0.529
Eggs [g/d, M(IQR)]	36.26 (18.86, 52.80)	35.16 (18.52, 52.80)	37.56 (18.86, 52.80)	*z* = -0.466	0.641
Fish and shrimp [g/d, M(IQR)]	18.93 (8.33, 44.20)	17.86 (8.33, 36.54)	21.43 (8.33, 44.64)	*z* = -1.009	0.313
Oils [g/d, M(IQR)]	44.44 (33.33, 50.52)	45.83 (34.58, 54.52)	41.67 (33.33, 50.00)	*z* = 0.680	0.497
Salt [g/d, M(IQR)]	6.67 (5.33, 8.33)	6.67 (5.56, 8.33)	6.46 (5.00, 8.33)	*z* = 1.868	0.062
Energy [kcal/d, M(SD)]	2964.96 (692.70)	2930.26 (669.37)	3000.40 (717.50)	*t* = -0.704	0.482
Protein [g/d, M(SD)]	92.78 (27.39)	91.29 (28.12)	94.31 (26.67)	*t* = -0.765	0.445
Fat [g/d, M(IQR)]	94.70 (74.00, 120.60)	91.85 (72.10, 116.85)	95.35 (74.90, 127.60)	*z* = -0.957	0.339
Carbohydrate [g/d, M(SD)]	424.67 (117.18)	424.18 (122.97)	425.16 (111.61)	*t* = -0.058	0.954
Log Dietary fiber [g/d, M(SD)]	1.20 (0.17)	1.17 (0.17)	1.23 (0.16)	*t* = -2.769	0.006
Cholesterol [mg/d, M(IQR)]	359.00 (224.75, 485.25)	347.50 (215.50, 484.50)	369.00 (242.50, 490.00)	*z* = -0.533	0.594
Log Vitamin A [μgRE/d, M(SD)]	2.82 (0.23)	2.79 (0.24)	2.84 (0.22)	*t* = -1.739	0.084
Thiamine [mg/d, M(SD)]	1.42 (0.39)	1.38 (0.37)	1.46 (0.41)	*t* = -1.367	0.173
Log Riboflavin [mg/d, M(SD)]	0.05 (0.16)	0.02 (0.16)	0.07 (0.16)	*t* = -2.059	0.041
Niacin [mg/d, M(SD)]	19.91 (5.90)	19.59 (6.07)	20.24 (5.75)	*t* = -0.768	0.443
Vitamin B_6_ [mg/d, M(IQR)]	0.41 (0.32, 0.52)	0.42 (0.31, 0.51)	0.41 (0.33, 0.54)	*z* = -0.719	0.472
Folate [μg/d, M(IQR)]	84.65 (59.05, 118.20)	78.45 (52.48, 96.90)	94.45 (71.13, 133.30)	*z* = -3.009	0.003
Log Vitamin C [mg/d, M(SD)]	2.10 (0.20)	2.05 (0.19)	2.15 (0.20)	*t* = -3.646	<0.001
Vitamin D [μg/d, M(IQR)]	1.50 (0.60, 3.28)	1.95 (0.68, 3.20)	1.25 (0.53, 3.30)	*z* = 0.668	0.504
Log Vitamin E [mg/d, M(SD)]	1.80 (0.13)	1.80 (0.13)	1.81 (0.13)	*t* = -0.880	0.380
Log Calcium [mg/d, M(SD)]	2.71 (0.19)	2.69 (0.19)	2.74 (0.19)	*t* = -1.857	0.065
Phosphorus [mg/d, M(SD)]	1431.01 (414.88)	1396.46 (403.20)	1466.29 (425.69)	*t* = -1.173	0.242
Log Potassium [mg/d, M(SD)]	3.39 (0.13)	3.37 (0.13)	3.41 (0.14)	*t* = -1.921	0.056
Log Sodium [mg/d, M(SD)]	3.58 (0.11)	3.60 (0.11)	3.57 (0.11)	*t* = 1.493	0.137

*M(SD), mean (standard deviation); M(IQR), median (interquartile range); Log, logarithmical. Natural-logarithmically transformed was performed on the values of dietary fiber, vitamin A, riboflavin, vitamin C, vitamin E, calcium, potassium and sodium.*

**TABLE 4:** Logistic regression analysis on the associations between quartiles of food and nutrients and vulnerable plaque (*N* = 194).

Variables	Univariate			Multivariable		
		
	Adjusted OR (95% CI)	*p*-value	*p* for trend	Adjusted OR (95% CI)	*p*-value	*p* for trend
*Model1: Food intakes*						
*Soy and nuts (g/d)*						
Q1 (0 [≤3.33])	1.00 (Reference)			1.00 (Reference)		
Q2 (7.14 [3.34–9.99])	0.58 (0.23, 1.48)	0.253		0.45 (0.14, 1.43)	0.177	
Q3 (15.47 [10.00–24.52])	0.53 (0.26, 1.11)	0.093		0.48 (0.20, 1.17)	0.105	
Q4 (50.00 [≥ 24.53])	0.41 (0.20, 0.87)	0.02	0.03	0.33 (0.13, 0.83)	0.019	0.028
*Vegetables (g/d)*						
Q1 ([194.17≤257.71])	1.00 (Reference)					
Q2 (298.63 [257.72–336.96])	0.75 (0.33, 1.69)	0.482				
Q3 (395.25 [336.97–471.20])	0.51 (0.23, 1.15)	0.106				
Q4 (548.64 [≥471.21])	0.32 (0.14, 0.73)	0.007	0.004			
*Fruits (g/d)*						
Q1 (27.33 [≤70.35])	1.00 (Reference)			1.00 (Reference)		
Q2 (116.15 [70.36–161.32])	1.17 (0.50, 2.73)	0.718		1.40 (0.52, 3.76)	0.505	
Q3 (219.10 [161.33–280.20])	0.28 (0.12, 0.65)	0.003		0.26 (0.10, 0.70)	0.007	
Q4 (345.37 [≥280.21])	0.27 (0.12, 0.62)	0.002	< *0*.001	0.37 (0.14, 0.99)	0.047	0.002
*Salt (g/d)*						
Q1 (4.50 [≤5.33])	1.00 (Reference)					
Q2 (6.25 [5.34–6.67])	2.96 (1.37, 6.38)	0.006				
Q3 (8.33 [6.68–8.33])	2.64 (1.22, 5.70)	0.013				
Q4 (10.00 [≥8.34])	2.98 (0.82, 10.83)	0.098	0.027			
*Model2: Nutrient intakes*						
*Dietary fiber (g/d)*						
Q1 (10.20 [≤12.1])	1.00 (Reference)					
Q2 (14.50 [12.2–16.4])	0.66 (0.29, 1.47)	0.307				
Q3 (17.95 [16.5–20.7])	0.53 (0.24, 1.20)	0.13				
Q4 (24.95 [≥20.8])	0.35 (0.15, 0.79)	0.012	0.011			
*Vitamin A (μgRE/d)*						
Q1 (366.00 [≤445.0])	1.00 (Reference)					
Q2 (562.00 [446.0–681.5])	1.05 (0.47, 2.35)	0.906				
Q3 (779.00 [681.6–886.0])	0.61 (0.28, 1.36)	0.227				
Q4 (1222.00 [≥887.0])	0.54 (0.24, 1.20)	0.129	0.070			
*Riboflavin (mg/d)*						
Q1 (0.73 [≤0.85])	1.00 (Reference)			1.00 (Reference)		
Q2 (0.99 [0.86–1.12])	0.59 (0.26, 1.33)	0.207		0.36 (0.10, 1.33)	0.127	
Q3 (1.29 [1.13–1.50])	0.43 (0.19, 0.96)	0.038		0.75 (0.17, 3.28)	0.700	
Q4 (1.75 [≥1.51])	0.52 (0.23, 1.17)	0.115	0.111	3.11 (0.48, 20.28)	0.236	0.074
*Folate (μg/d)*						
Q1 (41.90 [≤59.3])	1.00 (Reference)					
Q2 (76.00 [59.4–84.7])	1.25 (0.55, 2.82)	0.591				
Q3 (96.45 [84.8–118.2])	0.81 (0.37, 1.79)	0.608				
Q4 (148.10 [≥118.3])	0.32 (0.14, 0.74)	0.008	0.003			
*Vitamin C (mg/d)*						
Q1 (71.60 [≤99.8])	1.00 (Reference)			1.00 (Reference)		
Q2 (112.35 [99.9–130.4])	0.81 (0.36, 1.85)	0.618		0.35 (0.12, 1.02)	0.054	
Q3 (149.40 [130.5–182.1])	0.43 (0.19, 0.98)	0.044		0.23 (0.07, 0.74)	0.014	
Q4 (211.75 [≥182.2])	0.24 (0.10, 0.56)	0.001	< *0*.001	0.10 (0.03, 0.38)	0.001	0.003
*Calcium (mg/d)*						
Q1 (313.00 [≤387.0])	1.00 (Reference)			1.00 (Reference)		
Q2 (453.50 [388.0–524.5])	0.89 (0.40, 1.99)	0.77		2.52 (0.66, 9.62)	0.176	
Q3 (590.00 [524.6–696.0])	0.61 (0.27, 1.36)	0.226		1.29 (0.26, 6.36)	0.753	
Q4 (849.00 [≥697.0])	0.45 (0.20, 1.02)	0.055	0.036	0.41 (0.06, 2.59)	0.342	0.082
*Potassium (mg/d)*						
Q1 (1728.40 [≤2052.4])	1.00 (Reference)					
Q2 (2253.60 [2052.5–2473.0])	0.89 (0.40, 1.98)	0.768				
Q3 (2808.60 [2473.1–3057.7])	0.72 (0.32, 1.60)	0.419				
Q4 (3486.70 [≥3057.8])	0.54 (0.24, 1.20)	0.129	0.107			
*Sodium (mg/d)*						
Q1 (2851.00 [≤3231.0])	1.00 (Reference)			1.00 (Reference)		
Q2 (3455.50 [3232.0–3921.0])	1.33 (0.60, 2.96)	0.479		1.16 (0.42, 3.20)	0.769	
Q3 (4225.00 [3922.0–4623.0])	1.00 (0.45, 2.22)	1		0.70 (0.26, 1.92)	0.488	
Q4 (5233.50 [≥4624.0])	1.87 (0.84, 4.20)	0.127	0.198	2.98 (1.06, 8.37)	0.038	0.111

*Backward elimination method was applied to avoid multicollinearity. OR (95% CI) are presented with correction. The above model was adjusted for gender, age, body mass index, history of myocardial infarction, low-density lipoprotein cholesterol (LDL-C), total cholesterol (TC) and triglyceride (TG). OR, odds ratio; CI, confidence interval; Q, quartile.*

The OR between the greatest and lowest levels of soy and nut intake was 0.33 (95% CI = 0.13–0.83). Fruits were associated with 0.26 times (95% CI = 0.10–0.70) decreased risk of incident vulnerable plaques when comparing moderate (Q3 level) to low-consumers, this association was slightly weakened in the highest consumers (Q4 level, OR = 0.37, 95% CI = 0.14–0.99). Vitamin C intake was found to be inversely associated with the development of susceptible plaques, with OR of 0.10 (95% CI = 0.03–0.38) and 0.23 (95% CI = 0.07–0.74) in the highest (Q4) and moderate (Q3) levels, respectively, compared to the lowest level. When compared to low-consumers of sodium, high-consumers had a considerably higher risk of susceptible plaques (OR = 2.98, 95% CI = 1.06–8.37; all *p* < 0.05).

### Correlation Analysis Among the Variables

**FIGURE 4:**
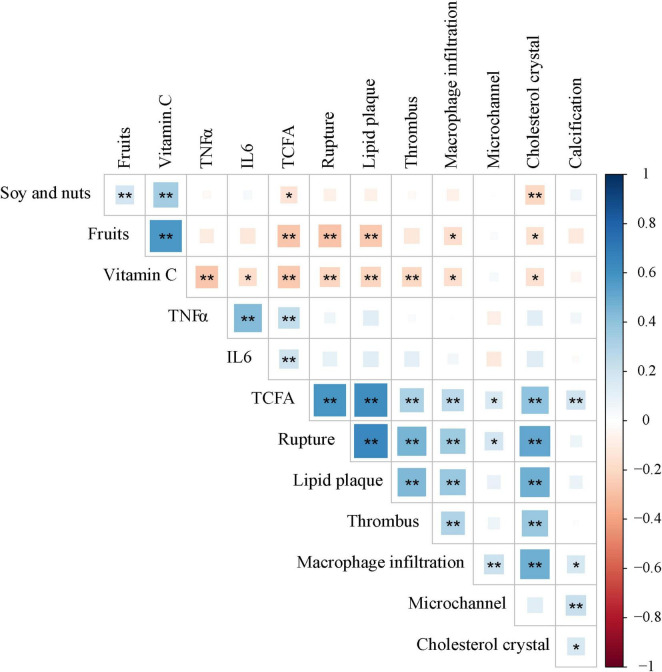
Correlations between soy and nuts, fruits and Vitamin C nutrients, Inflammatory factors and Plaque characteristics. *p < 0.05; **p < 0.01. TNF-α, tumor necrosis factor-α; IL-6, interleukin-6; TCFA, thin cap fibroatheroma.

As shown in Figure 4, vitamin C had negative correlations with TNF-α (*r* = −0.274, *p* < 0.001), IL-6 (*r* = −0.172, *p* = 0.016) and TCFA (*r* = −0.270, *p* < 0.001), rupture (*r* = −0.216, *p* = 0.002), lipid plaque (*r* = −0.217, *p* = 0.002), thrombus (*r* = −0.205, *p* = 0.004), macrophage infiltration (*r* = −0.173, *p* = 0.016), cholesterol crystal (*r* = −0.168, 0.020). Soy and nuts were negatively correlated with TCFA (*r* = −0.149, *p* = 0.037) and cholesterol crystal (*r* = −0.202, *p* = 0.005), and fruits were associated with rupture (*r* = −0.286, *p* < 0.001), lipid plaque (*r* = −0.267, *p* < 0.001), macrophage infiltration (*r* = −0.172, *p* = 0.017) and cholesterol crystal (*r* = −0.161, *p* = 0.024). Furthermore, TNF-α and IL-6 were positively correlated with TCFA (*r* = 0.250, *p* < 0.001 and *r* = 0.202, *p* = 0.005, respectively). However, there was no significant correlation between soy and nuts, fruits and TNF-α, IL-6.

### Mediation of the Relationship Between Vitamin C Consumption and TCFA by Inflammation Biomarkers

**FIGURE 5:**
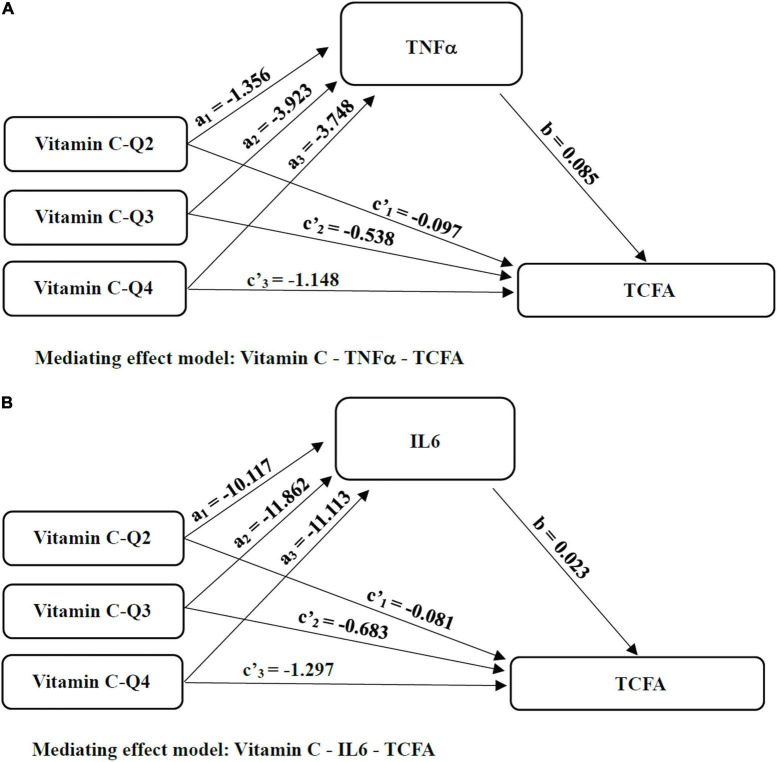
The mediated pathways between Vitamin C and TCFA. **(A)** Mediating effect model: Vitamin C-TNFα-TCFA; **(B)** Mediating effect model: Vitamin C-IL6-TCFA; Considering that the independent variable vitamin C as a multi-category variable divided by quartiles of continuous data, we sequentially coded the independent variable vitamin C and analyzed the mediating effect of the category variable with the minimum intake as reference. TNF-α, tumor necrosis factor-α; IL-6, interleukin-6; TCFA, thin cap fibroatheroma; Q, quartile.

To see if inflammation biomarkers (TNF-α and IL-6) mediated the effect of vitamin C on TCFA, we used SPSS Macro mediating analysis (Figure 5). In the mediation analysis, age, gender, BMI, history of myocardial infarction, TG, TC, and LDL-C were all used as covariates. The indirect effects (path *a* × *b*) of TNF-α significantly mediated the effects of vitamin C intake Q3 level (95% CI = −0.742, −0.072) and Q4 level (95% CI = −0.734, −0.064) on TCFA when the lowest consumer level (Q1) of vitamin C was used as the reference (Table 5). The 95% Bootstrap confidence interval of Q3 (*a*_2_ = −3.923, *b* = 0.085, *a*_2_*b* = −0.335) and Q4 (*a*_3_ = −3.748, *b* = 0.085, *a*_2_*b* = −0.320) levels relative to Q1 level excluded zero, indicating significant relative mediating effect (Figure 5A and Table 5). That is, the Q3 vitamin C consuming group produced 3.923 (*a*_2_ = −3.923, *p* < 0.01) less TNF-α than the Q1 level group, so the incidence of TCFA was correspondingly reduced (*b* = 0.085, *p* = 0.020). The vitamin C consumption group with Q4 level reduced TNF-α by 3.748 (*a*_3_ = −3.748, *p* < 0.01) compared with Q1 level group, resulting in lower TCFA (*b* = 0.085, *p* = 0.020). The relative direct effect was significant (95% CI = −2.029, −0.266), indicating that the risk of TCFA decreased by 1.148 (*c*’_3_ = −1.148, *p* = 0.011) in the group with Q4 level of vitamin C intake, after excluding the mediating effect (Table 5). And the same is true for IL-6. Taking the lowest intake group as a reference, IL-6 played a relative indirect mediating role in the effects of Q2 (95% CI = −0.788, −0.010), Q3 (95% CI = −0.875, −0.030) and Q4 (95% CI = −0.825, −0.010) levels of vitamin C consumption on TCFA (Table 5). Increased vitamin C intake with Q2 (*a*_1_ = −10.117, *b* = 0.023, *a*_1_*b* = −0.236), Q3 (*a*_2_ = −11.862, *b* = 0.023, *a*_2_*b* = −0.276) and Q4 (*a*_3_ = −11.113, *b* = 0.023, *a*_3_*b* = −0.259) level was associated with a decrease in serum levels of inflammatory factors, and furthermore, serum inflammatory factors levels mediated the beneficial effects of vitamin C on vulnerable plaques (Figure 5B and Table 5). In the meantime, the vitamin C consumption with Q4 level had relative direct effects on TCFA after controlling for IL-6 (*c*’_3_ = −1.297, *p* = 0.004, 95% CI = −2.168, −0.426; Table 5).

**TABLE 5:** Effect of Vitamin C on TCFA mediated by TNF-α and IL-6.

Independent variable	Mediators	Relative direct effects (*c*’)				Relative indirect effects (a*b)			
			
		Coeffect	SE	LLCI	ULCI	Effect	BootSE	LLCI	ULCI
*Vitamin C*	TNF-α								
Q2 (112.35 [99.9–130.4])		–0.097	0.433	–0.946	0.752	–0.116	0.128	–0.468	0.056
Q3 (149.40 [130.5–182.1])		–0.538	0.436	–1.393	0.317	–0.335	0.173	–0.742	–0.072
Q4 (211.75 [≥182.2])		–1.148	0.450	–2.029	–0.266	–0.320	0.174	–0.734	–0.064
*Vitamin C*	IL-6								
Q2 (112.35 [99.9–130.4])		–0.081	0.430	–0.923	0.762	–0.236	0.195	–0.788	–0.010
Q3 (149.40 [130.5–182.1])		–0.683	0.426	–1.517	0.151	–0.276	0.217	–0.875	–0.030
Q4 (211.75 [≥182.2])		–1.297	0.444	–2.168	–0.426	–0.259	0.207	–0.825	–0.010

The direct effect shows the direct relationship between Vitamin C and TCFA via path c’ when the mediator is included in the model. The indirect effect shows the indirect relationship between Vitamin C and TCFA via the mediator (i.e., path a*b). The indirect (mediation) effect is significant if the bootstrapped confidence intervals do not include 0. TNF-α, tumor necrosis factor-α; IL-6, interleukin-6; TCFA, thin cap fibroatheroma; Q, quartile; LLCI, Limited liability confidence interval; ULCI, Upper liability confidence interval.

## Discussion

Our analysis indicated that soy and nuts, fruits, and vitamin C were revealed to be dietary protective factors for plaque vulnerability, whereas sodium was found to be a dietary risk factor for plaque vulnerability. Vitamin C intake was found to be negatively related to IL-6 and TNF-α, whereas serum IL-6 and TNF-α were found to be favorably connected to vulnerable plaque. More importantly, TNF-α and IL-6 mediated the effects of vitamin C on vulnerable plaque, which was the first study to our knowledge to examine the mediating effects of inflammatory variables on the link between vitamin C and coronary plaque susceptibility using OCT. The findings of this study reveal that an elevated inflammatory response may explain the link between diet and plaque vulnerability in CHD patients, implying that a vitamin C-rich diet may help to reduce vulnerable plaque progression by lowering the inflammatory response.

Major advancements in coronary artery disease prevention have required early diagnosis of susceptible plaques in recent decades. A vulnerable plaque is a coronary atherosclerotic plaque that is prone to rupture, and TCFA is the most common phenotype of vulnerable plaque today. Large necrotic lipid core with an overlying thin fibrous cap (<65 μm) infiltrated by macrophages characterizes TCFA as the antecedent lesion of plaque rupture (43). TCFA and vulnerable plaques related features can be identified and evaluated by OCT, this permits *in vivo* detection of macrophages, intimal vasculature, and structures suggestive of calcium accumulation within atherosclerotic plaques, as well as plaque rupture, fibrous cap thickness, and intraluminal thrombus (44). This study by OCT exhibited that vulnerable plaque characteristics, including thinner fibrous cap, more prominent lipid arc, longer lipid core length and TCFA, were seen more frequently compared with non-vulnerable plaque (all *p* < 0.01). Meanwhile, the incidence of rupture, lipid plaque, macrophage infiltration, thrombus, calcification and cholesterol crystal in vulnerable plaques was fundamentally higher than that in non-vulnerable plaques (all *p* < 0.01), and huge contrast was also found in the incidence of microchannel (*p* = 0.022; Table 2). The natural history of coronary atherosclerosis has been shown to be a dynamic process ranging from early lesion development to more advanced plaques complicated by acute rupture and thrombosis; all of these indicators play key roles in the mechanisms of vulnerable plaque formation and rupture, according to studies. However, in cardiovascular illness, the production and disruption of susceptible plaque, followed by thrombosis, is regarded to be a crucial event that leads to acute coronary syndrome (ACS) and sudden cardiac death (43). As a result, physicians would be able to identify individuals at high risk for ACS and catastrophic CHD by detecting susceptible plaque *in vivo*.

Diet and eating habits have been demonstrated to have a significant impact on the occurrence and progression of heart disease, the world’s leading cause of mortality. Healthy eating habits play a crucial influence in the primary and secondary prevention of cardiovascular disease, according to meta-analysis studies (45). RCTs and observational studies both revealed an inverse relationship between adherence to good dietary patterns, particularly the Mediterranean diet, and cardiovascular risk, including major cardiovascular events (myocardial infarction, stroke, or death from cardiovascular causes) (16, 46). Diets low in whole grains, vegetables, fruits, and nuts but high in sodium were the leading dietary patterns for mortality, according to the latest systematic study of dietary patterns across 195 countries (47). Our findings back up the idea that a low intake of fruits and soy/nuts in the diet is linked to susceptible plaque, and that increasing the intake of cardioprotective food groups (soy/nuts, fruit) in real life is linked to improvements in plaque features in CHD patients. When compared to the lowest-consumers, consumption of ≥ 24.53 g/d (median value 50.00 g/d) of legumes and nuts from daily diet was related with a significantly lower incidence of susceptible plaque (OR = 0.33, 95% CI = 0.13–0.83, *p* for trend = 0.028). When fruits consumption climbed to 219.10 (161.33–280.20) g/d in CHD patients, a substantial protective impact against susceptible plaques was seen (OR = 0.26, 95% CI = 0.10–0.70, *p* = 0.007), and this positive effect exhibited a linear trend with the rise of fruits intake (*p* for trend = 0.002). Our statistically significant findings corroborated previous research, bolstering previous conclusions.

When compared to a low-fat diet, the PREDIMED research found that a Mediterranean diet rich in nuts could reduce CHD end points by about 30% (hazard ratio: 0.72, 95% CI: 0.54, 0.95) (46). The large levels of unsaturated fats, soluble fiber, plant protein, vitamins, minerals, and phytochemicals in nuts are likely to be responsible for their health benefits. Nuts high in long-chain n-3 polyunsaturated fatty acids (PUFAs) and omega-3 PUFAs may reduce plasma TGs and platelet aggregation, as well as have anti-inflammatory properties, which may reduce CHD risk and prevent coronary plaque progression. Nuts would also have provided phytosterols (which are known to inhibit cholesterol absorption and enhance fecal cholesterol excretion) and polyphenols (which are known antioxidants) (48). The high fiber and antioxidant content of legumes may explain their cardiovascular advantages. After 2 months of ingestion, a combination of soluble fiber and soy protein has been shown to drastically lower LDL-C by 20% (49). Increases in legume intake (25 g/d) were connected to increases in paraoxonase-1 activity (PON1, a crucial HDL-bound antioxidant enzyme) and decreases in cholesteryl ester transfer protein (CETP, pro-atherogenic when excessively active) activity, according to Hernáez et al. (13). These mechanisms could explain why increasing consumption of dietary legumes and nuts was linked to a lower risk of susceptible plaque. This is the first time, to our knowledge, that the effect of increasing legume and nut consumption on coronary atherosclerotic susceptible plaque risk in humans has been reported.

The evidence to date strongly suggests that increasing fruit consumption may lower the risk of CHD incidence and mortality. According to a meta-analysis, increasing fruit intake by 200 g per day reduces CHD risk by 10%. Globally, 710,000 CHD deaths were attributed to a fruit and vegetable consumption of less than 500 g per day, and this number grew to 1.34 million CHD deaths when 800 g per day was used as the recommended intake (10). The current study also found an inverse relationship between fruit intake and plaque vulnerability in CHD patients, implying that fruits were dietary preventive factors against plaque vulnerability. According to extant research, fruits and vegetables’ preventive mechanisms may include not only some of the recognized bioactive nutrient benefits based on antioxidant, anti-inflammatory, and electrolyte capabilities, but also their functional features, such as low glycemic load and energy density (50). As a result, it’s critical to concentrate on the impact of fruits and vegetables, as well as probable mechanisms of action and dietary recommendations for lowering the risk of vulnerable plaque. Despite the fact that various reviews and meta-analyses of fruit and vegetable diets have found an inverse relationship between intake and CHD risk, epidemiological evidence for causality is mixed. Indeed, differences in the types of fruits and vegetables ingested by research populations, as well as whether cooked or raw veggies are preferred, may introduce heterogeneity. As a result, stratified analysis of fruits and vegetables separately could help to reduce heterogeneity. This study analysis for fruits and vegetables separately, which were limited to fresh vegetables and fruits, not fruit juices, dry fruits, frozen and canned. But results from this study did not find a significant benefit of increasing vegetables intake for plaque vulnerability prevention. This result may be related to the habit of eating raw vegetables with high-salt sauces in the selected subject samples. Heat processing, on the other hand, can increase the availability of some bioactive substances (such as lycopene from tomatoes and carotenoids from carrots) and convert folate polyglutamate in vegetables into monoglutamate, which is more bioavailable (51). In addition, the research center locations in the cold northeast of China maybe limits the variety of vegetables available. Fruits and vegetables should be consumed as part of a healthy diet. We should not only consider the daily intake of fruits and vegetables in future dietary guidelines, but also their nutrient content, as well as how they are prepared (storage) and consumed (cooking).

Many elements, including water soluble vitamins, fiber, sterols, and phytochemicals, are credited with the benefits of a diet rich in fruits and vegetables. Dietary vitamins have received the most attention in the literature. These vitamins are hypothesized to defend against reactive oxygen species (ROS), which contribute to atherogenesis by producing superoxide, and so present a potential method by which they may prevent CHD. Fruits (especially citrus fruits like guavas and oranges) are the primary source of these anti-oxidant vitamins, particularly vitamin C (52, 53). Low dietary vitamin C consumption has been linked to an elevated risk of CHD in women in studies (54). Vitamin C intake was found to be inversely related to all-cause and cardiovascular mortality in a prospective cohort analysis (9, 55). Antioxidant vitamin C intake lowered the incidence of poor cardiovascular events, according to a 22-year follow-up research. Low vitamin C consumption from fruits and vegetables was also found to predict intracoronary in-stent restenosis in Type D patients in a prior study (17). The current study also found an inverse relationship between food-derived vitamin C and plaque vulnerability in CHD patients. And the relationship showed a strongly linear, dose-response trend. We observed a 77% lower risk of vulnerable plaque in the Q3 level versus lowest quartile of vitamin C intake. The protective effect became more significant when intake increased to the Q4 level (median value 211.75 mg/d). It is worth mentioning that the median intake of vitamin C in Q2 to Q4 levels, is greater than the current Recommended Dietary Allowance (RDA) for adults in China (100 mg/d), the median intake of vitamin C in Q4 level is still greater than a proposed intake (PI) for Chinese adults (200 mg/d) which established for the purpose of preventing or reducing the risk of non-communicable diseases, so it would be necessary to consider a larger dose to get the protector effect in high-risk groups of CHD. From this, a diet of more than five daily servings of fresh fruits and vegetables is recommended to achieve an intake of 200 mg/d for vitamin C (56). Overall, these results support our previous research findings (18). In animal models and *in vitro* cell culture systems, the link between dietary vitamin C and atheroma formation has been investigated (57). The antioxidant hypothesis has affirmed the protective effects of vitamin C. Meanwhile, the lipid oxidation theory claims that dietary vitamin C can slow the progression of atherosclerosis by lowering the creation of oxidized LDL cholesterol (50). However, the PREDIMED trial found that eating vitamin C-rich fruits and vegetables lowers plasma levels of inflammatory biomarkers such hs-CRP, IL-6, and TNF-α, which are linked to distinct stages of atheroma plaque development (28). As a result, we believe that vitamin C derived mainly from antioxidants diet (fruits and vegetables) is a key mechanism for preventing the formation of vulnerable plaque.

Vitamin C consumption was found to be negatively related to IL-6, TNF-α and vulnerable plaque in this study, but TNF-α and IL-6 were positively connected to vulnerable plaque. To put it another way, we found an inverse dose–response relationship between vitamin C intake with inflammation and plaque vulnerability. Further mediation analysis revealed that IL-6 and TNF-α, were wholly or partially mediating these correlations between vitamin C and vulnerable plaque, implying that the effect of increasing dietary vitamin C intake on vulnerable plaque risk was related to lower levels of serum inflammatory markers. These findings show that the inflammatory pathway may play a role in vitamin C’s preventive actions in the development of vulnerable plaques. Inflammation is hypothesized to aid in the onset and progression of atherosclerosis, as well as the development of acute thrombotic problems (49). The infiltration and retention of LDL cholesterol in the artery wall, in particular, is a crucial initiating event that activates an inflammatory response and promotes the progression of atherosclerosis. Monocyte recruitment is aided by higher amounts of adhesion molecules and chemotactic factors, as well as the promotion of activation, differentiation, and proliferation processes, as well as macrophage immobilization in the inflammatory plaque. The macrophages are eventually converted into foam cells. M-CSF promotes the production of TNF-α and release of proinflammatory cytokines including IL-6 (58), which leads to tissue damage. Activated macrophages then produce inflammatory cytokines that can destabilize lesions and promote thrombus formation, all of which can eventually lead to plaque activation and rupture (59). We believe that vitamin C’s anti-inflammatory and immunomodulating effects are mediated by downregulation of leukocyte adhesion molecules, resulting in a decrease in proinflammatory interleukins IL-6, TNF-α, and their receptors. In fact, inflammatory biomarkers appeared to be inversely associated to vegetable and fruit-based patterns and *a priori* healthy dietary patterns, as evidenced by intervention studies exploring the Mediterranean diet (28). Studies on the relationship between the dietary inflammatory index (DII, which measures the overall inflammatory impact of food) and cardiovascular diseases have shown that eating a better anti-inflammatory diet can help reduce the risk of CHD and related death (27, 60, 61). Previous research has also found a link between dietary inflammation and plaque susceptibility (60, 62, 63). All of these evidences back up the study’s findings.

Furthermore, this study discovered a favorable link between dietary sodium intake and plaque susceptibility. Subjects in the highest quartile progressed susceptible plaque 2.98 times greater than those in the lowest quartile. Previous research has found that as sodium intake rose, the prevalence of carotid atherosclerotic plaques increased (66 vs 90%, Tertile I vs Tertile III, *p* = 0.02) (64). According to the most recent meta-analysis, higher sodium consumption was linked to a higher risk of CHD (65). The current study also showed that salt intake has a negative impact on the formation of coronary atherosclerotic plaques. Dietary salt may affect endothelial function, as seen by increased matrix metalloproteinase-9 activity and serum CRP levels (64, 66, 67).

### Study Limitations

This study has certain limitations that should be acknowledged. To begin with, this was a single-center, cross-sectional mediation study with a limited sample size, which could result in selection bias. Second, because this was not a follow-up interventional trial, we are unable to establish causal correlations between variables. Third, while we included confounding variables that have been used in other CHD research, our analysis is confined to the common confounding variables that are typically addressed when investigating diet-CHD relationships. As a result, residual confounding from unmeasured variables cannot be ruled out. Finally, although it is convenient to collect data using SQFFQ, there may be recall bias in reporting dietary consumption based on subjects’ eating habits over the previous 12 months, including omission of food items and inaccurate nutrient consumption. In any case, large-scale multicenter prospective studies are needed to investigate the causal association between diet, inflammatory biomarkers, and susceptible plaque progression/regression, as well as to confirm the results of this investigation.

## Conclusion

In conclusion, the findings of this study add to the growing body of evidence supporting dietary prevention of poor CHD prognosis, suggesting that favoring cardioprotective food groups and nutrients (including soy and nuts, fruits and vitamin C) and limiting sodium intake help to reduce the likelihood of vulnerable plaque. More importantly, we also have demonstrated for the first time that inflammatory biomarkers IL-6 and TNF-α may mediate the relations between vitamin C and vulnerable plaque. This supports the hypothesis that vitamin C consumption could affect vulnerable plaque development through the inflammatory pathway. These new findings add to the underlying processes by which dietary categories and nutrients may influence CHD outcomes by adding another layer of regulation. CHD patients with suboptimal diet especially those in low vitamin C intake, may be potential beneficiaries of anti-inflammatory therapy. Our findings highlight the relevance of a high-quality food-based diet in general, and we provide a suggestion for a better diet to support the complementary therapeutic therapy of CHD patients at high risk for vulnerable plaques.

## Data Availability Statement

The raw data supporting the conclusions of this article will be made available by the authors, without undue reservation.

## Ethics Statement

The studies involving human participants were reviewed and approved by Human Ethics Review Committee at Harbin Medical University (China). The patients/participants provided their written informed consent to participate in this study.

## Author Contributions

LL and ZZ have conducted investigation and written the original draft of the manuscript. YW and GL were involved in data curation and analysis. XG and BY participated in the OCT analysis and writing – review and editing. PL was responsible for project conception and project supervision. All authors have read and agreed to the published version of the manuscript.

## Conflict of Interest

The authors declare that the research was conducted in the absence of any commercial or financial relationships that could be construed as a potential conflict of interest.

## Publisher’s Note

All claims expressed in this article are solely those of the authors and do not necessarily represent those of their affiliated organizations, or those of the publisher, the editors and the reviewers. Any product that may be evaluated in this article, or claim that may be made by its manufacturer, is not guaranteed or endorsed by the publisher.

## References

[1] Roth GA Mensah GA Johnson CO Addolorato G Ammirati E Baddour LM Global burden of cardiovascular diseases and risk factors, 1990–2019: update from the Gbd 2019 Study. *J Am Coll Cardiol.* (2020) 76:2982–3021. 10.1016/j.jacc.2020.11.010 33309175 PMC7755038

[2] Severino P D’Amato A Pucci M Infusino F Adamo F Birtolo LI Ischemic heart disease pathophysiology paradigms overview: from plaque activation to microvascular dysfunction. *Int J Mol Sci.* (2020) 21:1–30. 10.3390/ijms21218118 33143256 PMC7663258

[3] Kubo T Tanaka A Ino Y Kitabata H Shiono Y Akasaka T. Assessment of coronary atherosclerosis using optical coherence tomography. *J Atheroscler Thromb.* (2014) 21:895–903. 25069815 10.5551/jat.25452

[4] Finn AV Nakano M Narula J Kolodgie FD Virmani R. Concept of vulnerable/unstable plaque. *Arterioscler Thromb Vasc Biol.* (2010) 30:1282–92. 10.1161/ATVBAHA.108.179739 20554950

[5] Arbab-Zadeh A Fuster V. The myth of the “vulnerable plaque”: transitioning from a focus on individual lesions to atherosclerotic disease burden for coronary artery disease risk assessment. *J Am Coll Cardiol.* (2015) 65:846–55. 10.1016/j.jacc.2014.11.041 25601032 PMC4344871

[6] Stone GW Maehara A Ali ZA Held C Matsumura M Kjoller-Hansen L Percutaneous coronary intervention for vulnerable coronary atherosclerotic plaque. *J Am Coll Cardiol.* (2020) 76:2289–301. 10.1016/j.jacc.2020.09.547 33069847

[7] Pan A Lin X Hemler E Hu FB. Diet and cardiovascular disease: advances and challenges in population-based studies. *Cell Metab.* (2018) 27:489–96. 10.1016/j.cmet.2018.02.017 29514062 PMC5844273

[8] Lee CH Chan RSM Wan HYL Woo YC Cheung CYY Fong CHY Dietary intake of anti-oxidant vitamins a, C, and E is inversely associated with adverse cardiovascular outcomes in Chinese-a 22-years population-based prospective study. *Nutrients.* (2018) 10:3–11. 10.3390/nu10111664 30400367 PMC6265686

[9] Aune D Keum N Giovannucci E Fadnes LT Boffetta P Greenwood DC Dietary intake and blood concentrations of antioxidants and the risk of cardiovascular disease, total cancer, and all-cause mortality: a systematic review and dose-response meta-analysis of prospective studies. *Am J Clin Nutr.* (2018) 108:1069–91. 10.1093/ajcn/nqy097 30475962 PMC6250988

[10] Aune D Giovannucci E Boffetta P Fadnes LT Keum N Norat T Fruit and vegetable intake and the risk of cardiovascular disease, total cancer and all-cause mortality-a systematic review and dose-response meta-analysis of prospective studies. *Int J Epidemiol.* (2017) 46:1029–56. 10.1093/ije/dyw319 28338764 PMC5837313

[11] Jayedi A Zargar MS. Intake of vitamin B6, folate, and vitamin B12 and risk of coronary heart disease: a systematic review and dose-response meta-analysis of prospective cohort studies. *Crit Rev Food Sci Nutr.* (2019) 59:2697–707. 10.1080/10408398.2018.1511967 30431328

[12] Reynolds A Mann J Cummings J Winter N Mete E Te Morenga L. Carbohydrate quality and human health: a series of systematic reviews and meta-analyses. *Lancet.* (2019) 393:434–45. 10.1016/s0140-6736(18)31809-9 30638909

[13] Hernaez A Sanllorente A Castaner O Martinez-Gonzalez MA Ros E Pinto X Increased consumption of virgin olive oil, nuts, legumes, whole grains, and fish promotes Hdl functions in humans. *Mol Nutr Food Res.* (2019) 63:e1800847. 10.1002/mnfr.201800847 30628167

[14] Zhong VW Van Horn L Cornelis MC Wilkins JT Ning H Carnethon MR Associations of dietary cholesterol or egg consumption with incident cardiovascular disease and mortality. *JAMA.* (2019) 321:1081–95. 10.1001/jama.2019.1572 30874756 PMC6439941

[15] Bechthold A Boeing H Schwedhelm C Hoffmann G Knuppel S Iqbal K Food groups and risk of coronary heart disease, stroke and heart failure: a systematic review and dose-response meta-analysis of prospective studies. *Crit Rev Food Sci Nutr.* (2019) 59:1071–90. 10.1080/10408398.2017.1392288 29039970

[16] Stewart RAH Wallentin L Benatar J Danchin N Hagström E Held C Dietary patterns and the risk of major adverse cardiovascular events in a global study of high-risk patients with stable coronary heart disease. *Eur Heart J.* (2016) 37:1993–2001. 10.1093/eurheartj/ehw125 27109584 PMC4929377

[17] Wang Y Zhang S Zhang G Yu B Gao X Dai Z Association between type D personality and in-stent restenosis in patients treated with percutaneous coronary intervention: a mediation analysis of dietary patterns. *J Psychosom Res.* (2020) 138:110244. 10.1016/j.jpsychores.2020.110244 33002810

[18] Wang W Wang Y Gao X Zhao Z Li L Yu B Association between food and nutrients intakes and coronary plaque vulnerability in patients with coronary heart disease: an optical coherence tomography study. *Nutr Metab Cardiovasc Dis.* (2021) 31:201–8. 10.1016/j.numecd.2020.08.027 33268215

[19] Koganti S Karanasos A Regar E Rakhit RD. Association of systemic inflammatory biomarkers with morphological characteristics of coronary atherosclerotic plaque by intravascular optical coherence tomography. *Hellenic J Cardiol.* (2021) 62:101–6. 10.1016/j.hjc.2020.06.008 32628997

[20] Koyama K Yoneyama K Mitarai T Ishibashi Y Takahashi E Kongoji K Association between inflammatory biomarkers and thin-cap fibroatheroma detected by optical coherence tomography in patients with coronary heart disease. *Arch Med Sci.* (2015) 11:505–12. 10.5114/aoms.2015.52352 26170842 PMC4495146

[21] Eltoft A Arntzen KA Wilsgaard T Mathiesen EB Johnsen SH. Interleukin-6 is an independent predictor of progressive atherosclerosis in the carotid artery: the tromso study. *Atherosclerosis.* (2018) 271:1–8. 10.1016/j.atherosclerosis.2018.02.005 29453087

[22] Ammirati E Moroni F Norata GD Magnoni M Camici PG. Markers of inflammation associated with plaque progression and instability in patients with carotid atherosclerosis. *Mediators Inflamm.* (2015) 2015:718329. 10.1155/2015/718329 25960621 PMC4415469

[23] Barbaresko J Koch M Schulze MB Nothlings U. Dietary pattern analysis and biomarkers of low-grade inflammation: a systematic literature review. *Nutr Rev.* (2013) 71:511–27. 10.1111/nure.12035 23865797

[24] Bordoni A Danesi F Dardevet D Dupont D Fernandez AS Gille D Dairy products and inflammation: a review of the clinical evidence. *Crit Rev Food Sci Nutr.* (2017) 57:2497–525. 10.1080/10408398.2014.967385 26287637

[25] Almeida-de-Souza J Santos R Lopes L Abreu S Moreira C Padrao P Associations between fruit and vegetable variety and low-grade inflammation in portuguese adolescents from labmed physical activity study. *Eur J Nutr.* (2018) 57:2055–68. 10.1007/s00394-017-1479-y 28616763

[26] Chai W Morimoto Y Cooney RV Franke AA Shvetsov YB Le Marchand L Dietary red and processed meat intake and markers of adiposity and inflammation: the multiethnic cohort study. *J Am Coll Nutr.* (2017) 36:378–85. 10.1080/07315724.2017.1318317 28628401 PMC5540319

[27] Shivappa N Godos J Hébert J Wirth M Piuri G Speciani A Dietary inflammatory index and cardiovascular risk and mortality—a meta-analysis. *Nutrients.* (2018) 10:200. 10.3390/nu10020200 29439509 PMC5852776

[28] Casas R Urpi-Sardà M Sacanella E Arranz S Corella D Castañer O Anti-inflammatory effects of the mediterranean diet in the early and late stages of atheroma plaque development. *Mediators Inflamm.* (2017) 2017:1–12. 10.1155/2017/3674390 28484308 PMC5412172

[29] Miller SJ Batra AK Shearrer GE House BT Cook LT Pont SJ Dietary fibre linked to decreased inflammation in overweight minority youth. *Pediatr Obes.* (2016) 11:33–9. 10.1111/ijpo.12017 25728000

[30] Gibson R Eriksen R Chambers E Gao H Aresu M Heard A Intakes and food sources of dietary fibre and their associations with measures of body composition and inflammation in UK adults: cross-sectional analysis of the airwave health monitoring study. *Nutrients.* (2019) 11:1839. 10.3390/nu11081839 31398891 PMC6722677

[31] Figueiredo D Azevedo A Pereira M Barros HD. Definition of hypertension: the impact of number of visits for blood pressure measurement. *Rev Port Cardiol.* (2009) 28:775–83. 19894656

[32] Havel RJ. Classifications of the hyperlipidemias. *Annu Rev Med.* (1977) 28:195–209. 10.1146/annurev.me.28.020177.001211 193431

[33] American Diabetes Association. Classification and diagnosis of diabetes: standards of medical care in diabetes-2021. *Diabetes Care.* (2021) 44:S15–33. 10.2337/dc21-S002 33298413

[34] Tong EK Strouse R Hall J Kovac M Schroeder SA. National survey of U.S. health professionals’ smoking prevalence, cessation practices, and beliefs. *Nicotine Tob Res.* (2010) 12:724–33. 10.1093/ntr/ntq071 20507899 PMC6281036

[35] Dufour MC. What is moderate drinking? Defining “drinks” and drinking levels. *Alcohol Res Health.* (1999) 23:5–14. 10890793 PMC6761695

[36] Zhao WH Huang ZP Zhang X He L Willett W Wang JL Reproducibility and validity of a Chinese food frequency questionnaire. *Biomed Environ Sci.* (2010) 23:1–38. 10.1016/s0895-3988(11)60014-720486429

[37] Tian J Ren X Vergallo R Xing L Yu H Jia H Distinct morphological features of ruptured culprit plaque for acute coronary events compared to those with silent rupture and thin-cap fibroatheroma. *J Am Coll Cardiol.* (2014) 63:2209–16. 10.1016/j.jacc.2014.01.061 24632266

[38] Kume T Okura H Yamada R Kawamoto T Watanabe N Neishi Y Frequency and spatial distribution of thin-cap fibroatheroma assessed by 3-vessel intravascular ultrasound and optical coherence tomography: an ex vivo validation and an initial in vivo feasibility study. *Circ J.* (2009) 73:1086–91. 10.1253/circj.cj-08-0733 19359816

[39] Prati F Regar E Mintz GS Arbustini E Di Mario C Jang IK Expert review document on methodology, terminology, and clinical applications of optical coherence tomography: physical principles, methodology of image acquisition, and clinical application for assessment of coronary arteries and atherosclerosis. *Eur Heart J.* (2010) 31:401–15. 10.1093/eurheartj/ehp433 19892716

[40] Tearney GJ Regar E Akasaka T Adriaenssens T Barlis P Bezerra HG Consensus standards for acquisition, measurement, and reporting of intravascular optical coherence tomography studies: a report from the international working group for intravascular optical coherence tomography standardization and validation. *J Am Coll Cardiol.* (2012) 59:1058–72. 10.1016/j.jacc.2011.09.079 22421299

[41] Hayes AF *Introduction to Mediation, Moderation, and Conditional Process Analysis: A Regression-based Approach.* New York, NY: The Guilford Press (2013). p. 77.

[42] Preacher KJ Hayes AF. Asymptotic and resampling strategies for assessing and comparing indirect effects in multiple mediator models. *Behav Res Methods.* (2008) 40:879–91. 10.3758/brm.40.3.879 18697684

[43] Toutouzas K Benetos G Karanasos A Chatzizisis YS Giannopoulos AA Tousoulis D. Vulnerable plaque imaging: updates on new pathobiological mechanisms. *Eur Heart J.* (2015) 36:3147–54. 10.1093/eurheartj/ehv508 26419623

[44] Tian J Hou J Xing L Kim SJ Yonetsu T Kato K Significance of intraplaque neovascularisation for vulnerability: optical coherence tomography study. *Heart.* (2012) 98:1504–9. 10.1136/heartjnl-2012-302445 22869676

[45] Chareonrungrueangchai K Wongkawinwoot K Anothaisintawee T Reutrakul S. Dietary factors and risks of cardiovascular diseases: an umbrella review. *Nutrients.* (2020) 12:1088. 10.3390/nu12041088 32326404 PMC7231110

[46] Estruch R Ros E Salas-Salvado J Covas MI Corella D Aros F Primary prevention of cardiovascular disease with a mediterranean diet supplemented with extra-virgin olive oil or nuts. *N Engl J Med.* (2018) 378:e34. 10.1056/NEJMoa1800389 29897866

[47] Afshin A Sur PJ Fay KA Cornaby L Ferrara G Salama JS Health effects of dietary risks in 195 countries, 1990–2017: a systematic analysis for the global burden of disease study 2017. *Lancet.* (2019) 393:1958–72. 10.1016/s0140-6736(19)30041-8 30954305 PMC6899507

[48] Tapsell LC Neale EP Satija A Hu FB. Foods, nutrients, and dietary patterns: interconnections and implications for dietary guidelines. *Adv Nutr.* (2016) 7:445–54. 10.3945/an.115.011718 27184272 PMC4863273

[49] Torres N Guevara-Cruz M Velazquez-Villegas LA Tovar AR. Nutrition and atherosclerosis. *Arch Med Res.* (2015) 46:408–26. 10.1016/j.arcmed.2015.05.010 26031780

[50] Alissa EM Ferns GA. Dietary fruits and vegetables and cardiovascular diseases risk. *Crit Rev Food Sci Nutr.* (2017) 57:1950–62. 10.1080/10408398.2015.1040487 26192884

[51] Melse-Boonstra A West CE Katan MB Kok FJ Verhoef P. Bioavailability of heptaglutamyl relative to monoglutamyl folic acid in healthy adults. *Am J Clin Nutr.* (2004) 79:424–9. 10.1093/ajcn/79.3.424 14985217

[52] Sharma S Sheehy T Kolonel L. Sources of vegetables, fruits and vitamins a, C and E among five ethnic groups: results from a multiethnic cohort study. *Eur J Clin Nutr.* (2014) 68:384–91. 10.1038/ejcn.2013.271 24398639 PMC4933301

[53] Jia X Wang Z Zhang B Su C Du W Zhang J Food sources and potential determinants of dietary vitamin c intake in Chinese adults: a cross-sectional study. *Nutrients.* (2018) 10:320. 10.3390/nu10030320 29518947 PMC5872738

[54] Kubota Y Iso H Date C Kikuchi S Watanabe Y Wada Y Dietary intakes of antioxidant vitamins and mortality from cardiovascular disease: the Japan collaborative cohort study (Jacc) study. *Stroke.* (2011) 42:1665–72. 10.1161/STROKEAHA.110.601526 21512181

[55] Zhao LG Shu XO Li HL Zhang W Gao J Sun JW Dietary antioxidant vitamins intake and mortality: a report from two cohort studies of Chinese adults in Shanghai. *J Epidemiol.* (2017) 27:89–97. 10.1016/j.je.2016.10.002 28142039 PMC5363781

[56] Carr AC Lykkesfeldt J. Discrepancies in global vitamin c recommendations: a review of Rda criteria and underlying health perspectives. *Crit Rev Food Sci Nutr.* (2021) 61:742–55. 10.1080/10408398.2020.1744513 32223303

[57] Frikke-Schmidt H Lykkesfeldt J. Role of marginal vitamin C deficiency in atherogenesis: in vivo models and clinical studies. *Basic Clin Pharmacol Toxicol.* (2009) 104:419–33. 10.1111/j.1742-7843.2009.00420.x 19489786

[58] Mangge H Becker K Fuchs D Gostner JM. Antioxidants, inflammation and cardiovascular disease. *World J Cardiol.* (2014) 6:462–77. 10.4330/wjc.v6.i6.462 24976919 PMC4072837

[59] Calder P Albers R Antoine J Zhao J. Inflammatory disease processes and interactions with nutrition. *Br J Nutr.* (2009) 101:S1–45. 10.1017/S0007114509377867 19586558

[60] Peng M Wang L Xia Y Tao L Liu Y Huang F High dietary inflammatory index is associated with increased plaque vulnerability of carotid in patients with ischemic stroke. *Stroke.* (2020) 51:2983–9. 10.1161/strokeaha.120.029035 32921261

[61] Graffouillere L Deschasaux M Mariotti F Neufcourt L Shivappa N Hebert JR Prospective association between the dietary inflammatory index and mortality: modulation by antioxidant supplementation in the Su.Vi.Max randomized controlled trial. *Am J Clin Nutr.* (2016) 103:878–85. 10.3945/ajcn.115.126243 26864363 PMC4763501

[62] Bondonno NP Lewis JR Blekkenhorst LC Shivappa N Woodman RJ Bondonno CP Dietary inflammatory index in relation to sub-clinical atherosclerosis and atherosclerotic vascular disease mortality in older women. *Br J Nutr.* (2017) 117:1577–86. 10.1017/s0007114517001520 28673375

[63] Wei T Liu J Zhang D Wang X Li G Ma R The relationship between nutrition and atherosclerosis. *Front Bioeng Biotechnol.* (2021) 9:635504. 10.3389/fbioe.2021.635504 33959594 PMC8094392

[64] Mazza E Ferro Y Lamprinoudi T Gazzaruso C Doldo P Pujia A Relationship between high sodium and low Pufa intake and carotid atherosclerosis in elderly women. *Exp Gerontol.* (2018) 108:256–61. 10.1016/j.exger.2018.05.004 29747013

[65] Milanlouei S Menichetti G Li Y Loscalzo J Willett WC Barabasi ALA. Systematic comprehensive longitudinal evaluation of dietary factors associated with acute myocardial infarction and fatal coronary heart disease. *Nat Commun.* (2020) 11:6074. 10.1038/s41467-020-19888-2 33247093 PMC7699643

[66] Ferreira-Sae MC Cipolli JA Cornelio ME Matos-Souza JR Fernandes MN Schreiber R Sodium intake is associated with carotid artery structure alterations and plasma matrix metalloproteinase-9 upregulation in hypertensive adults. *J Nutr.* (2011) 141:877–82. 10.3945/jn.110.135921 21430243

[67] Khayyatzadeh SS Kazemi-Bajestani SMR Bagherniya M Mehramiz M Tayefi M Ebrahimi M Serum high C reactive protein concentrations are related to the intake of dietary macronutrients and fiber: findings from a large representative Persian population sample. *Clin Biochem.* (2017) 50:750–5. 10.1016/j.clinbiochem.2017.03s.016 28336391

